# Chronic conditions and multimorbidity associated with institutionalization among Finnish community-dwelling older people: an 18-year population-based follow-up study

**DOI:** 10.1007/s41999-021-00535-y

**Published:** 2021-07-14

**Authors:** Anna Viljanen, Marika Salminen, Kerttu Irjala, Elisa Heikkilä, Raimo Isoaho, Sirkka-Liisa Kivelä, Päivi Korhonen, Tero Vahlberg, Matti Viitanen, Maarit Wuorela, Minna Löppönen, Laura Viikari

**Affiliations:** 1 Health Care Center, Municipality of Lieto, Hyvättyläntie 7, 21420 Lieto, Finland; 2 Unit of Geriatrics, Department of Clinical Medicine, Faculty of Medicine, Turku City Hospital, FI-20014 University of Turku, Kunnallissairaalantie 20, 20700 Turku, Finland; 3 Welfare Division, City of Turku, Yliopistonkatu 30, 20101 Turku, Finland; 4 Unit of Family Medicine, Department of Clinical Medicine, Faculty of Medicine, University of Turku and Turku University Hospital, 20014 Turku, Finland; 5 Unit of Clinical Chemistry, Department of Clinical Medicine, Faculty of Medicine, TYKSLAB, 20521 Turku, Finland; 6 Social and Health Care, City of Vaasa, Ruutikellarintie 4, 65101 Vaasa, Finland; 7 Division of Social Pharmacy, Faculty of Pharmacy, University of Helsinki, 00014 Helsinki, Finland; 8 Unit of Biostatistics, Department of Clinical Medicine, Faculty of Medicine, University of Turku, Turku, Finland; 9 Division of Clinical Geriatrics, Department of Neurobiology, Care Sciences and Society, Center for Alzheimer Research, Karolinska Institutet and Karolinska University Hospital, Huddinge, Stockholm, Sweden; 10 Social and Health Care for Elderly, City of Raisio, Sairaalakatu 5, 21200 Raisio, Finland

**Keywords:** Aged, Institutionalization, Multimorbidity, Multiple chronic conditions

## Abstract

**Purpose:**

The ageing population is increasingly multimorbid. This challenges health care and elderly services as multimorbidity is associated with institutionalization. Especially dementia increases with age and is the main risk factor for institutionalization. The aim of this study was to assess the association of chronic conditions and multimorbidity with institutionalization in home-dwelling older people, with and without dementia.

**Methods:**

In this prospective study with 18-year follow-up, the data on participants’ chronic conditions were gathered at the baseline examination, and of conditions acquired during the follow-up period from the municipality’s electronic patient record system and national registers. Only participants institutionalized or deceased by the end of the follow-up period were included in this study. Different cut-off-points for multimorbidity were analyzed. Cox regression model was used in the analyses. Death was used as a competing factor.

**Results:**

The mean age of the participants (*n* = 820) was 74.7 years (64.0‒97.0). During the follow-up, 328 (40%) were institutionalized. Dementia, mood disorders, neurological disorders, and multimorbidity defined as five or more chronic conditions were associated with a higher risk of institutionalization in all the participants. In people without dementia, mood disorders and neurological disorders increased the risk of institutionalization.

**Conclusion:**

Having dementia, mood or neurological disorder and/or five or more chronic conditions were associated with a higher risk of institutionalization. These risk factors should be recognized when providing and targeting care and support for older people still living at home.

**Supplementary Information:**

The online version contains supplementary material available at 10.1007/s41999-021-00535-y.

## Key summary points

**Aim:**

The aim of the study is to assess the association of chronic conditions and multimorbidity with institutionalization in older people.

**Findings:**

Having dementia, mood or neurological disorder and/or five or more chronic conditions were associated with a higher risk of institutionalization.

**Message:**

These risk factors should be recognized in primary care when providing and targeting care and support for home-dwelling older people.

**Supplementary Information:**

The online version contains supplementary material available at 10.1007/s41999-021-00535-y.

## Introduction

In Finland, as in other Western countries, the population is ageing and the proportion of inhabitants aged 65 years or older is growing [1]. The proportion of dementia as a cause of death has increased during recent years [2] and dementia is also the leading cause of institutionalization in the elderly [3–5].

Other factors associated with a higher risk of institutionalization include higher age, living alone, low socioeconomic status, use of home care, low number of specialist visits, low self-rated health (SRH), low body mass index (BMI), cognitive and functional impairment including walking difficulties and falls, and several chronic conditions, such as Parkinson’s disease, mood disorders, stroke and multimorbidity [4–12]. Among the oldest old women (> 90 years), Parkinson’s disease, depression, hip fracture, and multimorbidity, in addition to dementia, predict a higher risk of institutionalization [13].

The majority of older people prefer to “age in place” as long as it is possible [14]. This is often also the municipality’s preferred choice as institutional care is expensive [15] and in Finland, most of it is paid for by the municipality. Institutionalization is increasingly concentrating to the last years of life [16]. The growing number of very old people with chronic conditions will lead to increased demand of care, especially institutional care [17, 18].

In research, multimorbidity is often defined by disease counts [5, 9, 13, 19] or weighed comorbidity indices, such as the Charlson Comorbidity Index (CCI) [20], and has been shown to predict mortality [19, 21] and institutionalization [5, 9, 13]. The definition of multimorbidity varies between studies. A systematic review suggests that the cut-off for multimorbidity, when using disease counts, should be selected by testing the number of conditions which best identify participants at higher risk of adverse outcomes [22].

The aim of this study was to assess the association of chronic conditions and multimorbidity with institutionalization among community-dwelling Finnish older people during an 18-year follow-up. We included also conditions acquired during the follow-up period in our analyses. Of interest were also these associations in people without dementia to discriminate which conditions primary care physicians should be aware of when assessing the risk of institutionalization of an older person without dementia.

## Methods

### Study design and population

This study is part of the longitudinal epidemiological study carried out in the municipality of Lieto in southwest Finland [23]. All persons born in or prior to the year 1933 (*n* = 1596) were invited to participate in the baseline examination that took place between March 1998 and September 1999. Of those eligible, 63 died before they were examined and 273 refused or did not respond leaving 1260 (82%) participants, 533 men and 727 women.

At baseline, the study protocol consisted of an extensive interview on demographic and socioeconomic factors and health behavior, numerous laboratory tests, and a clinical examination including a comprehensive survey of the participants’ medical records [23].

Participants already living in institutional care at baseline (*n* = 68) were excluded from the analyses. Also participants no longer living in Lieto at the end of 2016 (*n* = 86) were excluded from the analyses, as it was not possible to ascertain whether they continued living at home or were institutionalized in another municipality.

To ascertain the participants categorized as non-institutionalized were not institutionalized at a later date, we only included participants institutionalized or deceased by January 2017, leaving 820 participants. Also, because the aim of this study was to assess the association of chronic conditions acquired during the participants’ lifetime with institutionalization, we do not have the complete data on the acquired conditions of the participants who were still alive and living at home at the end of the follow-up period. The non-institutionalized participants include, therefore, only participants who deceased while living at home by the end of the follow-up period. The excluded participants still living at home in January 2017 (*n* = 286) were younger, more often women, more often living with someone than alone, had higher Mini-Mental State Examination (MMSE) scores and were less multimorbid than the study population (*n* = 820) (data not shown).

### Chronic conditions

The chronic conditions and their 10th revision of the International Statistical Classification of Diseases and Related Health Problems (ICD‒10) [24] codes considered in this study are shown in Online Resource 1. Systemic atrophies, extrapyramidal, and movement disorders (ICD-10: G10‒G26) are referred to hereafter as neurological disorders.

Data of chronic conditions were gathered at the baseline examination and from the municipality’s electronic patient record system and the official Finnish Care Register for Health Care including the Register of Primary Health Care Visits during the follow-up period.

### Multimorbidity

In this study, several cut-points for multimorbidity were used. Multimorbidity was defined as having three or more chronic conditions (multimorbidity3 +), four or more chronic conditions (multimorbidity4 +), five or more chronic conditions (multimorbidity5 +) or six or more chronic conditions (multimorbidity6 +).

### Institutionalization

Institutionalization was defined as permanent entry into a long-term care facility, of which the data were gathered from the municipality’s electronic patient record system and coded by month and year of entry.

### Statistical analyses

Differences in categorical baseline characteristics between the institutionalized and non-institutionalized participants were tested using the *χ*^2^ test. Mean ages between the two groups were compared with two-sample *t* test.

Hazard ratios (HRs) and their 95% confidence intervals (CI) for institutionalization were calculated using Cox proportional hazard models. The follow-up period was calculated from the baseline measurements to the institutionalization of the individual. We used death as a competitive factor in the analyses.

First, unadjusted Cox regression analyses were conducted for the association of chronic conditions and multimorbidity with institutionalization in the study population (*n* = 820). For the purpose of analyzing the association of chronic conditions and multimorbidity with institutionalization in participants without dementia, we excluded the participants with dementia (*n* = 334), which left us with 486 participants. Unadjusted Cox regression analyses were conducted for the association of chronic conditions and multimorbidity with institutionalization in participants without dementia.

Second, Cox regression analyses were adjusted for age, gender, living situation and MMSE scores. Third, unadjusted and adjusted multivariable analyses featuring variables found significantly associated with an increased risk of institutionalization in the adjusted analyses were conducted.

*P* values less than 0.05 were considered statistically significant. All statistical analyses were performed using SAS System for Windows, version 9.4 (SAS Institute Inc., Cary, NC, USA).

## Results

### Baseline characteristics

Baseline characteristics of the 820 participants are shown in Table 1. The participants institutionalized during the follow-up-period were older, more often women, more often living alone before institutionalization, and had lower MMSE scores at baseline than those not institutionalized. There were no differences in BMI levels, education, self-rated health, self-reported walking ability, having someone to help if needed or frailty by Frail Scale [25] between the groups (data not shown).

**Table 1:** Baseline characteristics of study participants according to institutionalization (*n* = 820)

	Institutionalized (*n* = 328)	Not institutionalized (*n* = 492)	*P* value
^a^Age, years	75.3 (6.5) [64‒95]	74.2 (6.9) [64‒97]	0.008
	*n* (%)	*n* (%)	
Age, years			0.073
64–74	163 (50)	284 (58)	
75–84	134 (41)	166 (34)	
≥ 85	31 (9)	42 (9)	
Gender			< 0.001
Men	105 (32)	252 (51)	
Women	223 (68)	240 (49)	
Living			0.001
Alone	140 (43)	153 (31)	
With someone	188 (57)	339 (69)	
^b^MMSE			0.002
≥ 26	247 (75)	413 (84)	
< 26	81 (25)	79 (16)	

Baseline examination between March 1998 and September 1999

^a^Values are mean (standard deviation) [range]

^b^Mini-Mental State Examination

### Follow-up characteristics

Of the 820 participants, 328 (40%) were institutionalized during the follow-up period of 18 years (Table 2). A significantly larger proportion of institutionalized participants had dementia, mood disorders, neurological disorders, and hypothyroidism than those not institutionalized. A significantly smaller proportion of institutionalized participants had malignant neoplasms, ischemic heart disease, atrial fibrillation, atherosclerosis, chronic lower respiratory diseases, and renal failure than those not institutionalized.

**Table 2:** Participants according to chronic conditions, multimorbidity and institutionalization (*n* = 820)

Chronic conditions	Institutionalized (*n* = 328) *n* (%)	Not institutionalized (*n* = 492) *n* (%)	*P* value
Malignant neoplasms (except basal cell carcinomas)	74 (23)	212 (43)	< 0.001
Iron deficiency anaemia	39 (12)	57 (12)	0.894
B12-vitamin anaemia	23 (7)	40 (8)	0.556
Hypothyroidism	47 (14)	48 (10)	0.045
Diabetes mellitus	78 (24)	125 (25)	0.597
Hypercholesterolaemia	114 (35)	174 (35)	0.858
Dementia	230 (70)	104 (21)	< 0.001
Mood disorders	150 (46)	104 (21)	< 0.001
^a^Systemic atrophies, extrapyramidal and movement disorders	40 (12)	30 (6)	0.002
Hypertension	198 (60)	290 (59)	0.684
Ischemic heart disease	169 (52)	302 (61)	0.005
Atrial fibrillation	98 (30)	184 (37)	0.026
Intracranial haemorrhage	17 (5)	23 (5)	0.740
Stroke	124 (38)	159 (32)	0.105
Atherosclerosis	23 (7)	54 (11)	0.057
Chronic lower respiratory diseases	62 (19)	124 (25)	0.035
Renal failure	33 (10)	77 (16)	0.021
^b^Multimorbidity3 +	293 (89)	406 (83)	0.007
^b^Multimorbidity4 +	244 (74)	333 (68)	0.039
^b^Multimorbidity5 +	168 (51)	213 (43)	0.026
^b^Multimorbidity6 +	96 (29)	116 (26)	0.068

^a^Referred to as neurological disorders in the main text

^b^The number signifies the number of chronic conditions used as a cut-off for multimorbidity; multimorbidity3 + denotes three or more chronic conditions, multimorbidity4 + denotes four or more chronic conditions, multimorbidity5 + denotes five or more chronic conditions and multimorbidity6 + denotes six or more chronic conditions

**Fig. 1:**
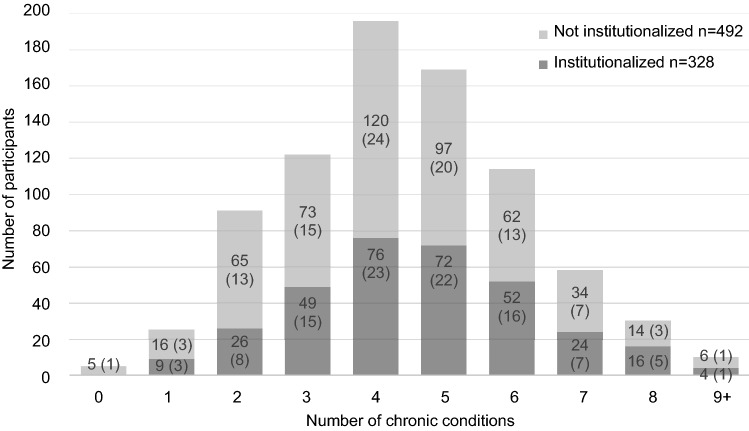
Participants (not institutionalized and institutionalized) according to their number of chronic conditions (*n* = 820). Percentages of participants with each number of chronic conditions in either category [not institutionalized (*n* = 492) or institutionalized (*n* = 328)] are shown in parentheses

The study population was very multimorbid (Fig. 1). A significantly larger proportion of institutionalized participants had multimorbidity3 + , multimorbidity4 + , and multimorbidity5 + than those not institutionalized.

Of the institutionalized participants, 230 (70%) had dementia (Table 3). Among the institutionalized participants without dementia (IPWOD), there was a significantly higher proportion of malignant neoplasms than among the institutionalized participants with dementia (IPWD). The proportion of participants with mood disorders was high (46%) in both groups. Multimorbidity defined all the four ways was more common among the IPWD than among the IPWOD.

**Table 3:** Institutionalized participants according to dementia and other chronic conditions (*n* = 328)

Chronic condition	With dementia (*n* = 230) *n* (%)	Without dementia (*n* = 98) *n* (%)	*P* value
Malignant neoplasms (except basal cell carcinomas)	42 (18)	32 (33)	0.004
Iron deficiency anaemia	24 (10)	15 (15)	0.212
B12-vitamin anaemia	14 (6)	9 (9)	0.315
Hypothyroidism	37 (16)	10 (10)	0.164
Diabetes mellitus	50 (22)	28 (29)	0.183
Hypercholesterolaemia	86 (37)	28 (29)	0.125
Mood disorders	105 (46)	45 (46)	0.965
^a^Systemic atrophies, extrapyramidal and movement disorders	23 (10)	17 (17)	0.063
Hypertension	136 (59)	62 (63)	0.484
Ischemic heart disease	120 (52)	49 (50)	0.718
Atrial fibrillation	66 (29)	32 (33)	0.474
Intracranial haemorrhage	10 (4)	7 (7)	0.296
Stroke	86 (37)	38 (39)	0.813
Atherosclerosis	13 (6)	10 (10)	0.140
Chronic lower respiratory diseases	41 (18)	21 (21)	0.446
Renal failure	23 (10)	10 (10)	0.955
^b^Multimorbidity3 +	212 (92)	81 (83)	0.011
^b^Multimorbidity4 +	178 (77)	66 (67)	0.018
^b^Multimorbidity5 +	128 (56)	40 (41)	0.005
^b^Multimorbidity6 +	76 (33)	20 (20)	0.007

^a^Referred to as neurological disorders in the main text

^b^The number signifies the number of chronic conditions used as a cut-off for multimorbidity; multimorbidity3 + denotes three or more chronic conditions, multimorbidity4 + denotes four or more chronic conditions, multimorbidity5 + denotes five or more chronic conditions and multimorbidity6 + denotes six or more chronic conditions

### Association of morbidity with institutionalization

In unadjusted analyses, dementia, mood disorders, neurological disorders, hypothyroidism, multimorbidity3 + , and multimorbidity5 + were significantly associated with a higher risk of institutionalization (Table 4). After adjustments, the association persisted in dementia, mood disorders, neurological disorders, and also multimorbidity5 + . Malignant neoplasms, ischemic heart disease, atrial fibrillation, and renal failure were significantly associated with a lower risk of institutionalization and the association persisted after adjustments.

**Table 4:** Association of chronic conditions and multimorbidity with institutionalization during the 18-year follow-up (*n* = 820)

Chronic conditions	All participants (*n* = 820)				Participants without dementia (*n* = 486)			
	Unadjusted HR (95% CI)	*P* value	^a^Adjusted HR (95% CI)	*P* value	Unadjusted HR (95% CI)	*P* value	^a^Adjusted HR (95% CI)	*P* value
Malignant neoplasms (except basal cell carcinomas)	0.46 (0.36‒0.60)	< 0.001	0.51 (0.40‒0.67)	< 0.001	0.58 (0.38‒0.89)	0.013	0.62 (0.40‒0.95)	0.029
Iron deficiency anaemia	1.01 (0.73‒1.41)	0.955	0.98 (0.70‒1.36)	0.900	1.23 (0.72‒2.09)	0.455	1.12 (0.65‒1.92)	0.681
B12-vitamin anaemia	0.88 (0.58‒1.35)	0.561	0.75 (0.48‒1.19)	0.229	1.01 (0.51‒1.99)	0.981	0.86 (0.41‒1.77)	0.674
Hypothyroidism	1.42 (1.04‒1.93)	0.030	1.21 (0.87‒1.70)	0.250	1.02 (0.54‒1.94)	0.953	0.82 (0.43‒1.59)	0.560
Diabetes mellitus	0.93 (0.72‒1.20)	0.585	0.95 (0.73‒1.23)	0.686	1.05 (0.68‒1.62)	0.825	1.10 (0.71‒1.70)	0.661
Hypercholesterolaemia	0.93 (0.75‒1.17)	0.540	0.88 (0.70‒1.12)	0.294	0.81 (0.52‒1.25)	0.333	0.72 (0.46‒1.12)	0.140
Dementia	4.84 (3.80‒6.16)	< 0.001	4.73 (3.69‒6.05)	< 0.001				
Mood disorders	2.31 (1.86‒2.87)	< 0.001	2.00 (1.58‒2.52)	< 0.001	2.92 (1.97‒4.34)	< 0.001	2.69 (1.77‒4.10)	< 0.001
^b^Systemic atrophies, extrapyramidal and movement disorders	1.71 (1.24‒2.37)	0.001	1.97 (1.41‒2.77)	< 0.001	2.74 (1.59‒4.72)	< 0.001	3.31 (1.85‒5.91)	< 0.001
Hypertension	0.97 (0.78‒1.22)	0.804	0.97 (0.76‒1.23)	0.781	1.04 (0.69‒1.58)	0.844	1.03 (0.66‒1.59)	0.913
Ischemic heart disease	0.75 (0.61‒0.93)	0.010	0.73 (0.58‒0.91)	0.005	0.65 (0.44‒0.96)	0.032	0.63 (0.43‒0.94)	0.025
Atrial fibrillation	0.75 (0.59‒0.95)	0.015	0.77 (0.60‒0.98)	0.030	0.76 (0.50‒1.16)	0.205	0.75 (0.49‒1.14)	0.181
Intracranial haemorrhage	1.07 (0.66‒1.74)	0.771	1.11 (0.68‒1.83)	0.672	1.48 (0.69‒3.16)	0.314	1.43 (0.67‒3.06)	0.351
Stroke	1.21 (0.96‒1.51)	0.101	1.14 (0.91‒1.45)	0.257	1.35 (0.90‒2.03)	0.146	1.35 (0.89‒2.03)	0.160
Atherosclerosis	0.68 (0.44‒1.04)	0.077	0.76 (0.49‒1.16)	0.199	1.04 (0.54‒1.99)	0.910	1.20 (0.62‒2.32)	0.591
Chronic lower respiratory diseases	0.75 (0.57‒1.00)	0.041	0.80 (0.60‒1.07)	0.127	0.82 (0.51‒1.32)	0.406	0.88 (0.54‒1.43)	0.616
Renal failure	0.65 (0.45‒0.92)	0.015	0.67 (0.47‒0.96)	0.028	0.58 (0.31‒1.11)	0.101	0.56 (0.29‒1.08)	0.084
^c^Multimorbidity3 +	1.50 (1.04‒2.16)	0.032	1.31 (0.90‒1.91)	0.165	1.11 (0.65‒1.90)	0.713	0.96 (0.55‒1.66)	0.879
^c^Multimorbidity4 +	1.25 (0.97‒1.60)	0.087	1.14 (0.87‒1.48)	0.340	1.08 (0.71‒1.65)	0.728	0.98 (0.64‒1.51)	0.917
^c^Multimorbidity5 +	1.26 (1.01‒1.56)	0.037	1.25 (1.00‒1.56)	0.0498	1.05 (0.70‒1.57)	0.802	1.05 (0.70‒1.58)	0.799
^c^Multimorbidity6 +	1.20 (0.95‒1.52)	0.120	1.19 (0.93‒1.52)	0.159	0.91 (0.56‒1.47)	0.685	0.90 (0.54‒1.47)	0.662

*HR* Hazard ratio, *CI* Confidence interval

^a^Adjusted for age, gender, living situation, and MMSE score

^b^Referred to as neurological disorders in the main text

^c^The number signifies the number of chronic conditions used as a cut-off for multimorbidity; multimorbidity3 + denotes three or more chronic conditions, multimorbidity4 + denotes four or more chronic conditions, multimorbidity5 + denotes five or more chronic conditions and multimorbidity6 + denotes six or more chronic conditions

In participants without dementia, mood disorders and neurological disorders were associated with a higher risk, and malignant neoplasms and ischemic heart disease with a lower risk of institutionalization in unadjusted and adjusted analyses.

Dementia, mood disorders, neurological disorders, malignant neoplasms, ischemic heart disease, atrial fibrillation, renal failure, and multimorbidity5 + were then included in a multivariable model. In unadjusted and adjusted multivariable analyses, dementia, mood disorders and neurological disorders were associated with an increased risk of institutionalization, and malignant neoplasms with a decreased risk of institutionalization (data not shown).

## Discussion

Dementia, mood disorders and neurological disorders, such as Parkinson’s disease, were associated with a significantly higher risk of institutionalization in an unselected community-dwelling population of older people, even after adjustments and in the multivariable analyses. These findings are in concordance with previous research [3–5, 9, 10, 13, 18, 26]. In our study, Parkinson’s disease dementia was included in the pooled dementia diagnosis (ICD‒10: F00‒F03, G30), but also separately, neurological disorders (including Parkinson’s disease) increased the risk of institutionalization. Hypothyroidism was associated with a higher risk of institutionalization in the unadjusted analyses. Thyroidal illnesses have also earlier been associated with a higher risk of institutionalization [5].

Previous research has found that in older individuals without dementia, higher age, living alone, functional and cognitive impairment, depression, stroke, diabetes, myocardial infarction, low SRH, and walking difficulties are associated with a higher risk of institutionalization [6, 27]. In this study, among participants without dementia, mood disorders were associated with a higher risk of institutionalization, a similar result to previous research [27]. Neurological diseases were also associated with a higher risk of institutionalization probably due to the induced functional impairment which has earlier been associated with institutionalization in individuals without dementia [6, 27].

In this study, multimorbidity3 + and multimorbidity5 + were associated with a higher risk of institutionalization in unadjusted analyses and multimorbidity5 + in adjusted analyses. In previous studies, a higher risk of institutionalization has been associated with multimorbidity defined as three or more, or four or more chronic conditions [9, 13]. In participants without dementia, multimorbidity was not associated with a higher risk of institutionalization in our study.

A recent systematic review and meta-analysis concluded that the most used cut-off for multimorbidity is two or more conditions, but it also suggested the possible approach of testing the number of conditions which best identify participants at higher risk of adverse effects [22]. Also, another systematic review on multimorbidity suggests the use of three or more chronic conditions as the definition of multimorbidity because using the classic definition of two or more conditions yields too many patients to be meaningful to clinicians [28]. For this reason, we analyzed the study population’s distribution of chronic conditions.

Our study population was very multimorbid, probably partly because we also accounted for the chronic conditions acquired during the follow-up period and not only baseline data, and also because the prevalence of multimorbidity increases with age [29] and has been increasing in the population of older people in recent years [30]. The highest variation in prevalence has been observed at the age of 75 years [28] and the prevalence of four or more chronic conditions has been increasing even more than the prevalence of two or three chronic conditions [30]. Thus, the use of only a cut-off of 2 or 3 or more chronic conditions for defining multimorbidity would not have been reasonable in our study population. In this study, only a substantial disease burden of five or more chronic conditions was associated with a higher risk of institutionalization. We suggest the cut-off for multimorbidity to be defined as 5 or more chronic conditions when assessing the risk of institutionalization in an unselected community-dwelling population of older people, especially when accounting for also the chronic conditions acquired during the follow-up period and not only the baseline information. The selection of 17 chronic conditions used in this study was in concordance with the CCI [20] and the simple primary care comorbidity index [19] and with the suggestion of using at least 12 conditions to choose from when assessing multimorbidity [28].

Multimorbidity poses a challenge to the health care system as it is simply not the sum of its parts and current disease specific guidelines seldom provide explicit guidance on how to treat patients with multiple conditions [31]. However, guidelines for treatment of multimorbidity are also emerging [32] as it has been recognized as the most common condition managed in clinical practice [33]. The main principles of managing patients with multimorbidity in primary care are a comprehensive approach and continuity and coordination of care [29, 34]. Older patients with multimorbidity need services that are flexible and focused on their individual situation, and often also need comprehensive geriatric assessment to timely target needs-based treatment and rehabilitation. However, the success of these efforts in this common but challenging patient group cannot only be evaluated by how many patients eventually are institutionalized as these interventions can also improve the situation of the older people continuing to live at home.

Malignant neoplasms, ischemic heart disease, atrial fibrillation, and renal failure were associated with a lower risk of institutionalization also in adjusted analyses and malignant neoplasms also in multivariable analyses, a similar result to earlier studies [3, 13]. However, another recent study found renal failure to be associated with a higher risk of institutionalization [5]. These conditions are associated with a higher risk of death when compared to healthy individuals, but they do not necessarily lead to such disabilities in daily life that might require institutional care before death. That probably explains the decreased risk of institutionalization for these chronic conditions in our study. Of these conditions, malignant neoplasms and ischemic heart disease were associated with a lower risk of institutionalization in unadjusted, and adjusted analyses, also in participants without dementia, somewhat contrary to a previous finding that having a myocardial infarction might increase the risk of institutionalization in participants without dementia [27].

Malignant neoplasms were associated with a lower risk of institutionalization but the higher prevalence among the IPWOD than among the IPWD might suggest that the participants in need of more complex palliative care may require institutional care at some point, although nowadays palliative care at home is common with help of the municipality’s at-home hospital.

The strengths of this study are the large sample size of an unselected community-dwelling population, high participation rate and the long follow-up period. We gathered the comprehensive information of the participant’s chronic conditions at baseline, and from baseline to the end of the follow-up period to study also the association of conditions acquired during the follow-up period with institutionalization, similarly as in previous studies [7, 8, 10, 27]. However, the dates of institutionalization were gathered from the electronic patient record system and are, therefore, more exact compared to these previous studies [7, 8, 10, 27]. We also used death as a competing factor in our analyses.

We included only participants that had been institutionalized or had died during the follow-up period to ascertain that we did not categorize participants who were still alive and living at home at the end of the follow-up as non-institutionalized when in fact they could have been institutionalized after the end of the follow-up period. This approach has been used before [27] but has not always been considered in earlier studies [7, 8, 10]. This approach is important when assessing also the association of conditions acquired during the follow-up period, and not only the association of baseline conditions with institutionalization. By omitting the participants still living at home at the end of the follow-up period, we also ascertained that we had complete data on the participants’ acquired conditions during their lifetime. The excluded participants were, however, in better health than the included participants and this explains why there were no differences in baseline variables of self-rated health, self-reported walking ability or frailty between the institutionalized and not institutionalized participants, contrary to what we found in our previous work [12].

The institutionalized participants were older, more often women, living alone at baseline, and had lower MMSE scores. These findings are in concordance with previous research [5, 7, 8, 17]. We, therefore, adjusted the analyses for these factors.

A limit to this study is that we categorized multimorbidity only by counting the chronic conditions and did not weigh the conditions according to their probability of inducing disability and thus institutionalization. Also, some of the conditions were considered in groups, for instance malignant neoplasms, and a participant could have had one or more of these conditions and it would have been counted as one. Our chosen chronic conditions also included conditions that when treated, should not have an impact on an individual’s risk of institutionalization, such as iron deficiency anaemia. However, our study sample, the number of chronic conditions considered and the definition of multimorbidity were in line with the study conduct suggested for multimorbidity studies [28], and by selecting the cut-off for multimorbidity to be higher than two or more chronic conditions, we probably diluted the effect of the less disabling conditions.

Institutionalization is of course a multifactorial process, not only influenced by the individual’s chronic conditions, but also by many socioeconomical factors, such as use of formal and informal home care [5, 26, 35], that were not considered here. Also, a simple diagnosis does not tell anything about the severity or induced disability of the condition, and these factors were not considered.

## Conclusions

Having dementia, a mood or neurological disorder, and/or having five or more chronic conditions were associated with a higher risk of institutionalization. These factors should be taken into account in primary care when assessing the future risk of institutionalization of an older person. The identified persons at a higher risk should be targeted by interventions to prevent or delay institutionalization.

## Supplementary Information

Below is the link to the electronic supplementary material.

Supplementary file1 (PDF 292 kb)

## Data Availability

The datasets used and/or analyzed during the current study are available from the corresponding author on reasonable request.
