# Supplementary material for: Chronic conditions and multimorbidity associated with institutionalization among Finnish community-dwelling older people: an 18-year population-based follow-up study
Source: Eur Geriatr Med. 2021 Jul 14;12(6):1275–84. doi: 10.1007/s41999-021-00535-y (PMC8626405; doi:10.1007/s41999-021-00535-y)
Supplement: Supplementary file 1 — Supplementary file1 (PDF 292 kb) [file 41999_2021_535_MOESM1_ESM.pdf]

Chronic conditions and multimorbidity associated with institutionalization among Finnish community-dwelling older people: an 18-year population-based follow-up study.

European Geriatric Medicine

Authors

MD Anna Viljanen · PhD Marika Salminen · MD, PhD Kerttu Irjala · MD Elisa Heikkilä · MD, Associate Professor Raimo Isoaho · Professor Sirkka-Liisa Kivelä · Professor Päivi Korhonen · MSc Tero Vahlberg · Professor Matti Viitanen · MD, PhD Maarit Wuorela · MD, PhD Minna Löppönen · MD, PhD Laura Viikari

Corresponding author: Anna Viljanen, University of Turku, Unit of Geriatrics, FI-20014 University of Turku, Finland. E-mail: amvilj@utu.fi.

Appendix 1 Chronic conditions and their [a]ICD-10 codes considered in this study

Chronic conditions	ICD-10
Malignant neoplasms (except basal cell carcinomas)	C0–C97 (except C44.01, C44.11, C44.21, C44.31, C44.41, C44.51, C44.61, C44.71, C44.81, C44.91)
Iron deficiency anaemia	D50
B12-vitamin anaemia	D51
Hypothyroidism	E03, E89
Diabetes mellitus	E10–E14
Hypercholesterolaemia	E78
Dementia	F00–F03, G30
Mood disorders	F30–F39
[b]Systemic atrophies, extrapyramidal and movement disorders	G10–G26
Hypertension	I10–I15
Ischemic heart disease	I20–I25
Atrial fibrillation	I48
Intracranial haemorrhage	I60–I62
Stroke	I63–I69, G45
Atherosclerosis	I70
Chronic lower respiratory diseases	J40–J47
Renal failure	N17–N19

[a]10th revision of the International Statistical Classification of Diseases and Related Health Problems

[b]Referred to as neurological disorders in the text
